# A new finding in the key prognosis-related proto-oncogene *FYN* in hepatocellular carcinoma based on the WGCNA hub-gene screening trategy

**DOI:** 10.1186/s12885-022-09388-5

**Published:** 2022-04-09

**Authors:** Chenkai Huang, Juanjuan Zhou, Yuan Nie, Guihai Guo, Anjiang Wang, Xuan Zhu

**Affiliations:** Department of Digestive Diseases, The First Affiliated Hospital of Nanchang University, Nanchang, 330006 Jiangxi China

**Keywords:** Hepatocellular carcinoma, Weighted gene coexpressed network analysis, FYN, Biomarker

## Abstract

**Background:**

Hepatocellular carcinoma (HCC) is the third-most deadly cancer worldwide. More breakthroughs are needed in the clinical practice for liver cancer are needed, and new treatment strategies are required. This study aims to determine the significant differences in genes associated with LIHC and further analyze its prognostic value further.

**Methods:**

Here, we used the TCGA-LIHC database and the profiles of GSE25097 from GEO to explore the differentially co-expressed genes in HCC tissues compared with paratumor (or healthy) tissues. Then, we utilized WGCNA to screen differentially co-expressed genes. Finally, we explored the function of FYN in HCC cells and xenograft tumor models.

**Results:**

We identified ten hub genes in the protein–protein interaction (PPI) network, but only three (*COLEC10, TGFBR3*, and *FYN*) appeared closely related to the prognosis. The expression of *FYN* was positively correlated with the prognosis of HCC patients. The xenograft model showed that overexpression of *FYN* could significantly inhibit malignant tumor behaviors and promote tumor cell apoptosis.

**Conclusion:**

Thus, *FYN* may be central to the development of LIHC and maybe a novel biomarker for clinical diagnosis and treatment.

**Supplementary Information:**

The online version contains supplementary material available at 10.1186/s12885-022-09388-5.

## Introduction

According to the 2019 data of the National Cancer Center [1], by the end of 2015, the number of malignant tumors nationwide was approximately 3.929 million, and the number of related deaths was approximately 2.338 million. Data show that, on average, more than 10,000 people are diagnosed with cancer every day, and 7.5 people are diagnosed with cancer every minute. Among cancer diagnoses, lung cancer, liver cancer, upper digestive system tumors, and colorectal cancer are the primary malignant tumors in China [2].

Liver cancer is known for its aggressiveness and high mortality rate. Most liver cancer patients in China were already in an advanced stage when diagnosed, and their treatment options are minimal [2, 3]. Additionally, due to accompanying blood metastasis and tumor thrombus formation, the five-year survival rate after liver cancer surgery in China is lower than that in developed countries in Europe and America [1]. Therefore, realizing the early diagnosis of liver cancer is particularly important for treating liver cancer and improving the prognoses [4]. At present, the most used clinical diagnostic marker for liver cancer is AFP [5]. However, the sensitivity and specificity of AFP in diagnosing liver cancer are not very satisfactory [6]. Therefore, we still need to identify additional biomarkers closely related to liver cancer progression to assist in clinical diagnosis and treatment.

WGCNA (weighted gene co-expression network analysis) aims to establish a multigene co-expression model to cluster similar gene expression similarly and analyze the relationship between differences and phenotypes within the module [7]. Compared with traditional methods, WGCNA establishes a more significant relationship between the gene mRNA expression and prognosis data. We combined WGCNA and differential gene expression analysis to further identify differential genes further, and these genes with significant correlations can be treated as candidate biomarkers [8–11].

Here we have screened out three hub genes (*TGFBR3*, *COLEC10*, and *FYN*) with potential value in clinical practice. As one of the Src family proteins and tumor suppressor genes, FYN has been little researched on liver cancer. Therefore, we explored the biological functions of FYN in liver cancer cells.

## Materials and Methods

### Data source

All gene expression data of LIHC (liver hepatocellular carcinoma) were obtained from the TCGA database (portal.gdc.cancer.gov/projects/TCGA-LIHC) and GEO database. Furthermore, LIHC data and corresponding clinical information were downloaded by R package *TCGAbiolinks* for free as previously described [12, 13]. The data comprised, 424 samples, including 374 LIHC tissues and 50 healthy tissues and RNAseq count data on 19,601 genes, and "Not Available (N/A)" to replace the missing values for further analysis. All genes with low abundance were excluded following the edgeR package recommendation.

Additionally, the R package *GEOquery* was used to normalize the expression profiles of GSE25097 from the GEO database (www.ncbi.nlm.nih.gov/geo/query/acc.cgi?acc=GSE25097). GSE25097 consisted of 268 tumor samples and 243 paired normal tissues from patients with LIHC and 6 healthy liver samples. As a result, approximately 2,887 genes were selected for further analysis.

### WGCNA Analysis

Gene expression data profiles of TCGA and GSE25097 were used to construct to gene co-expression networks in the WGCNA package in R to explore the modules of positively correlated genes among samples to relate modules to external sample traits. The adjacency was transformed into a TOM by using dissTOM. Co-expressed genes were aggregated into different MEs by the dynamic tree cut method. We determined on a cut line (< 0.25) for merging highly similar modules to make the modules more compact and the most significantly and correlated modules with LIHC were selected. Limma in R was applied to screen out DEGs. The DEGs in the TCGA-LIHC and GSE25097 datasets were visualized as a volcano plot using the R package ggplot2. Subsequently, potential prognostic genes were identified by overlapping genes between DEGs and co-expressed genes and were presented as a Venn diagram by VennDiagram in the R package.

### Functional Annotation and Construction of PPI and Screening of Hub Genes

The R package cluster profile package was used to explore the functions among genes of interest. GO annotation containing the three subontologies biological process, cellular component, and molecular function identified the natural properties of the genes and gene sets for all organisms. Then the STRING online tool was used to construct a PPI network of hub genes. The expression level of each hub gene was presented as a box plot graph.

### Cell Culture

Huh-7 and Hep3B cells were all purchased from ATCC (Manassas, USA). The Huh-7 cell culture medium consisted of DMEM and 10% FBS (Gibco, MA, USA); The Hep3B cell culture medium was MEM with nonessential non-essential amino acids (NEAAs) and 10% FBS.

### Reagents and Transfection

The FYN overexpression vector was purchased from BGI (Beijing, China). An FYN overexpression lentivirus was constructed by psPAX2 and pMD2.G plasmids in HEK293T, and its expression in HCC cells was verified by western blot analysis. According to the manufacturer's instructions, all the vectors and controls were transfected into cells using Lipofectamine 2000 (Invitrogen).

### Evaluation of HCC malignant behaviors

To obtain proliferation curves: HCC cells (Hep3B and Huh-7) were plated into 96-well plates at 2.5 × 10^3^/well and cultured in certain medium. The number of cells was determined using the CCK8 (Dojindo, Tokyo, Japan) every day for 7 days.

For the Transwell assay: cells in the logarithmic phase were digested and counted to prepare a cell suspension. The Transwell chamber was removed under sterile conditions and placed in a 24-well plate for use. Spread Matrigel gel (Matrigel collagen solution: DMEM (or MEM) medium 1: 3) in the Transwell cell used for invasion experiments to avoid air bubbles. Add 500 μL of complete medium to the 24-well plate and put the prepared Transwell chamber back into the dish to prevent air bubbles. A 200 μL aliquot of cell suspension was added to the Transwell cell; the number of cells in each cell is about 2.5 × 10^4^/L. The cells underwent continued cultivation for the corresponding times, after which the membrane was stained with 0.1% crystal violet and 20% methanol. Five fields of cells were used for further statistical analysis.

### Apoptotic Analysis of Terminal Dexynucleotidyl Transferase (Tdt)-Mediated dUTP Nick End Labeling (TUNEL) Staining

Cells were fixed with 4% paraformaldehyde after drug treatment for 30 min and washed three times with PBS. Then, 0.3% Triton X-100 was added, and the cells were incubated at room temperature for 5 min, and rinse three times with PBS. Prepare an appropriate amount of TUNEL test solution was prepared according to the TUNEL instructions and mixed thoroughly. Add (100 μL) was added of TUNEL detection solution to the sample, and the sample was incubated at 37 °C in the dark for and washed three times with phosphate-buffered saline (PBS). After mounting with anti-fluorescence quenching mounting solution, the sample was observed and photographed using a fluorescence microscope.

### Immunohistochemical Staining

For the dewaxing and hydration of sections, use 0.3% H_2_O_2_ was used to eliminate endogenous peroxidase, followed by antigen retrieval. The slides were incubated with primary antibodies overnight at 4 °C and incubated with a secondary antibody at room temperature for 30 min. Then use neutral gum was used to seal the sections. Images were collected, and five random images were selected for statistical analysis.

### Human HCC Xenograft Model

BALB/c nude mice (male, 5–6 weeks old) were purchased and maintained under SPF conditions with a 12 h on/off light cycle. About 1 × 10^6^ Huh-7 cells were injected into the right flank subcutaneously. The mice were randomly divided into two groups (8 animals each) when the tumors reached an average volume of approximately 60 mm^3^. Tumor volumes were calculated according to the following equation: Volume (mm^3^) = Length × (Width)^2^ × π/6. No animals were excluded, and feeding was continued for 1 week. The mice were sacrificed by cervical dislocation, and the tumor volumes and weights were measured.

### Statistical Analysis

**Fig. 1:**
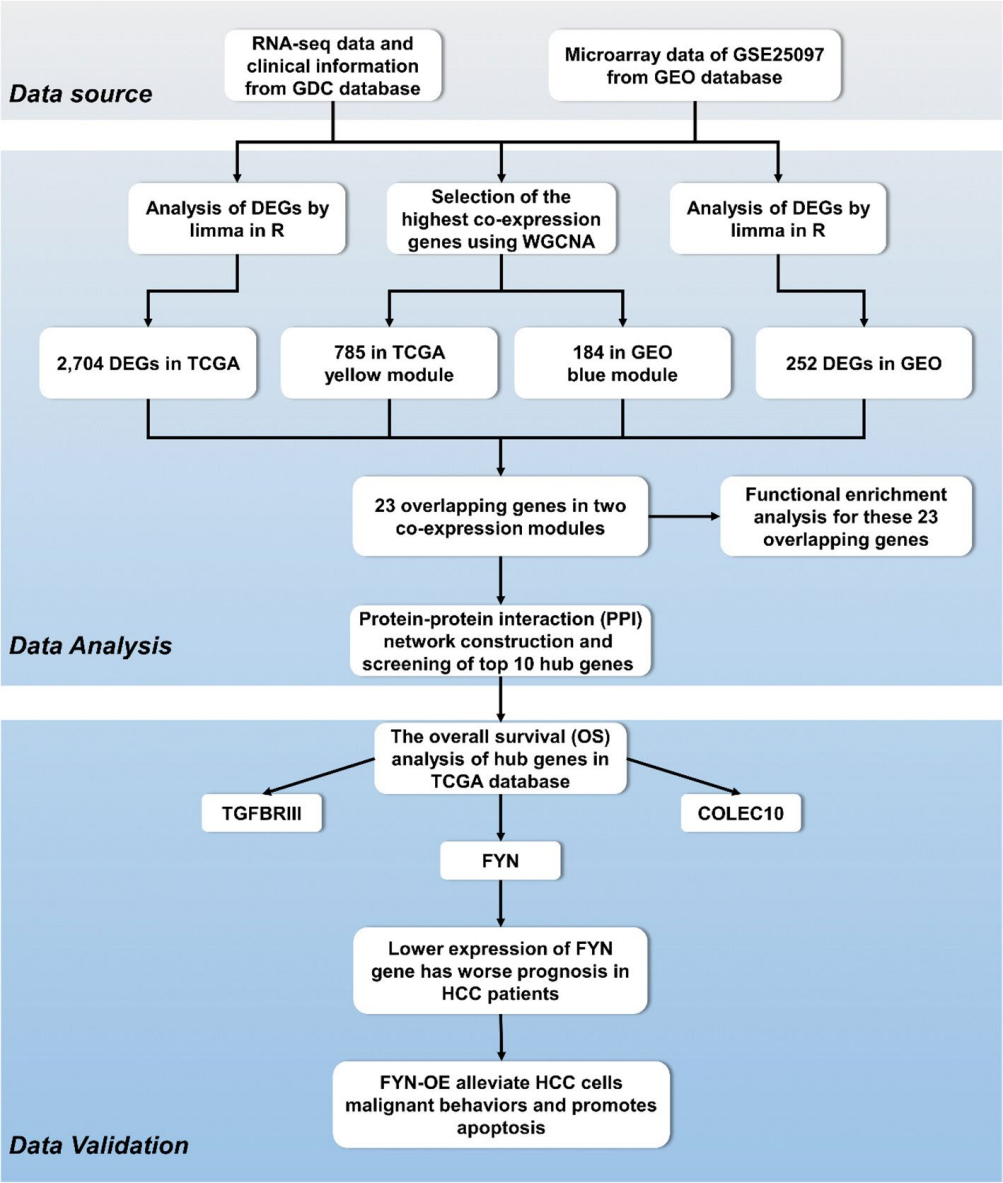
Study design and workflow for LIHC hub gene screening

The workflow of this study was shown is shown in Fig. 1. All statistical analyses were performed using SPSS V23.0 software. Comparisons of data with normal distribution between two groups were performed using Student's t-tests. Otherwise, Otherwise, comparisons were performed using the nonparametric Mann–Whitney test or the Wilcoxon matched-pairs test. Kaplan–Meier analysis and the log-rank test were used to compare the survival probabilities between different groups. Statistical tests were two-tailed, and a *P* < of 0.05 was considered statistically significant.

## Results

### Identification of Differentially Expressed Genes (DEGs) in Both TCGA and GEO database

**Fig. 2:**
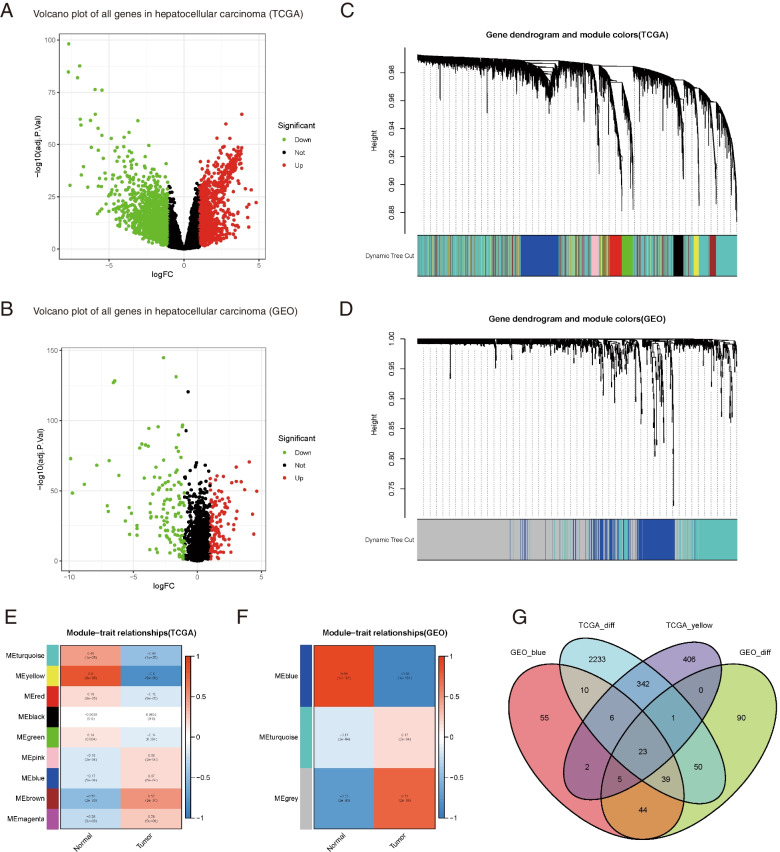
WGCNA modules associated with the clinical information in the TCGA-LIHC dataset. **A** **B** Volcano plot of all genes in hepatocellular carcinoma from TCGA and GEO. **C** **D** The hierarchical clustering of genes produced the cluster dendrogram of the co-expression network modules, and each module was assigned to one color, respectively. **E F** Relationships between tumor and normal tissues in TCGA-LIHC and GEO. Each row presents with a different color module, and the column corresponds to clinical trait (including HCC and non-HCC tissues) respectively. Each module contains the *P* value of corresponding correlation. **G** The Venn diagram among DEGs. About 23 overlapped genes are listed in the intersection of the DEG lists and two co-expression modules in total

For data collection, after analyses of the RNA-Sequencing and microarray data and GES25097 dataset to identify DEGs between LIHC and normal tissues, there were 2704 and 252 DEGs in TCGA and GES25097, respectively. The heatmap plots are shown in Supplementary Fig. 1 A, B. The volcano plot of the genes was shown in Fig. 2 A, B.

Subsequently, we identified candidate biomarkers and therapeutic targets to screen coexpression networks. This study constructed gene co-expression networks of the gene expression data profiles of TCGA-LIHC and GSE25097 using the WGCNA package in R (Fig. 2 C, D). By gathering similarly expressed genes in TCGA, a total of 9 modules was identified, whereas there are three modules in the GEO dataset (Fig. 2 E, F). The MEs in the TCGA yellow and GEO blue modules showed a higher correlation with the histologic grade of HCC. Therefore, we chose these two modules for further research.

### The Kyoto Encyclopedia of Genes and Genomes (KEGG) and Gene Ontology (GO) enrichment analyses of DEGs

**Fig. 3:**
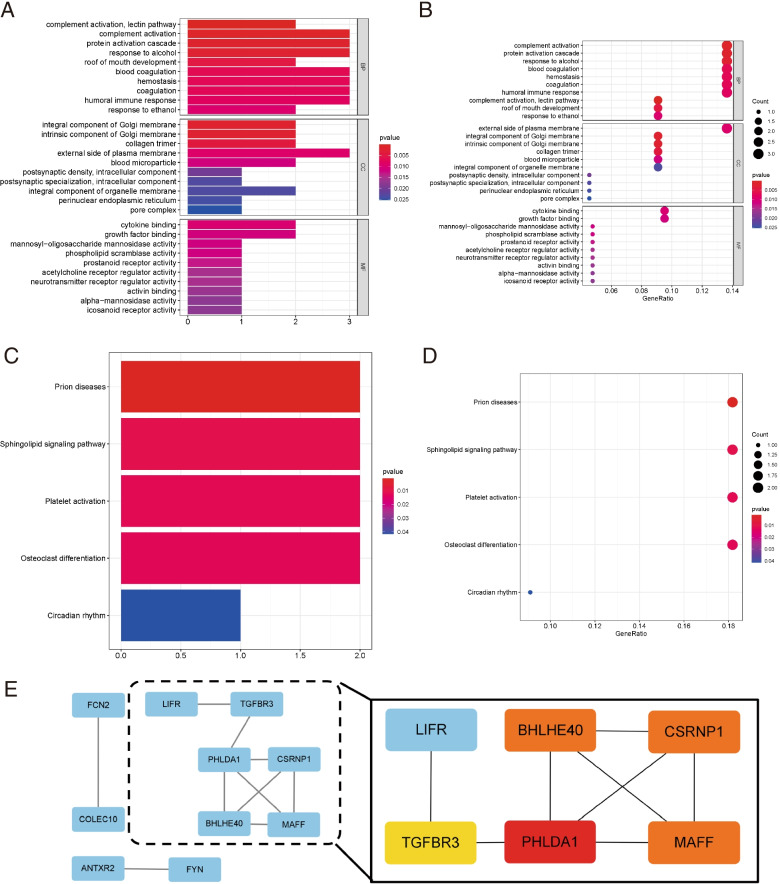
KEGG and GO enrichment analyses of all DEGs and the PPI network of hub-genes. **A** **B** KEGG and GO analyses of the TCGA-LIHC database. **C** **D** KEGG and GO analyses of the GEO dataset. The color represents the adjusted P-values, and the size of the spots represents the gene number. **E** The protein–protein interaction (PPI) network and the candidate hub genes. Edges represent the protein–protein associations. The red nodes represent genes with a high MCC score, while the yellow node represents genes with a low MCC score

We entered the DEGs into DAVID to analyze the significant GO and KEGG pathways [14]. GO annotation contained the three subontologies, biological process, cellular component, and molecular function, can identify the biological characteristics of all the organism's genes and gene sets (Fig. 3 A, C). We observed several enriched genes sets combined with the KEGG enrichment analysis shown in Fig. 3 B-D, including the complementary activation, protein activation cascade, and alcohol responding pathway in the TCGA database, prion disease, and sphingolipid signaling pathways in the GEO dataset.

### Identification of Hub Genes from DEGs

As shown in Fig. 2 G, in total, the 23 overlapping genes were extracted to validate the genes in the co-expression modules. Cytoscape was used to construct the PPI networks of all genes in the four modules. We used the "cytoHubba app" to calculate the degree of each node. Ten top hub-genes are showen in Fig. 3 E, among which *PHLDA1* seemed to act as the central role in the PPI network. Furthermore, all ten hub genes were subjected to further analysis.

### Verification of the Prognostic Values of the Hub Genes

**Fig. 4:**
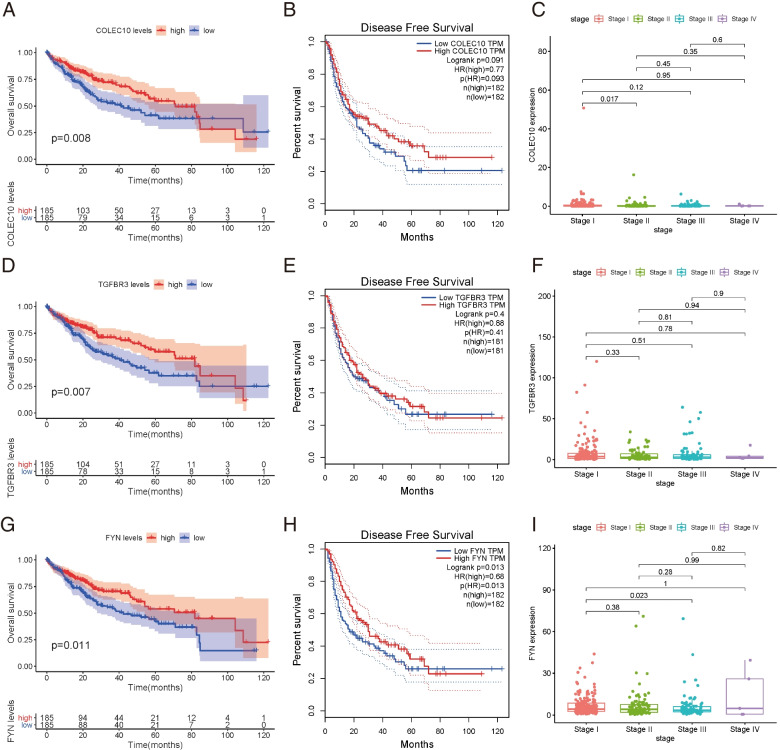
Survival analysis and disease-free survival (DFS) analysis of 3 hub genes in LIHC patients. **A** **B** Survival analysis for COLEC10 in LIHC. **C** COLEC10 expression in each stage in HCC patients. **D** **E** Survival analysis for TGFBR3 in LIHC. **F** TGFBR3 expression in each stage in HCC patients. **G** **H** Survival analysis for FYN in LIHC. **I** **F** FYN expression in each stage in HCC patients

To identify whether these hub genes have a prognostic value, the overall survival (OS) and disease-free survival (DFS) analyses of the ten hub genes were performed by Kaplan–Meier plotting using the GEPIA2 database R survival package. As shown in Fig. 4, *COLEC10*, *TGFBR3*, and *FYN*, three genes have a close relative to the survival rate of HCC patients. Among these three genes, a lower expression level of *FYN* was significantly associated with worse OS and DFS of the HCC patients (Fig. 4 G, H), while *CLOEC10* and *TGFBR3* were only associated with worse OS (Fig. 4 A, B, D, E). Unfortunately, both *COLEC10*, *TGFBR3*, and *FYN* expressions were not related to the staging phase of HCC patients (Fig. 4 C, F, I). Since the *FYN* gene is rarely studied in HCC, we will do further researched on *FYN* to clarify its role in liver cancer progression.

### Overexpression of *FYN* Inhibited the Malignant Behaviors of HCC Cells

**Fig. 5:**
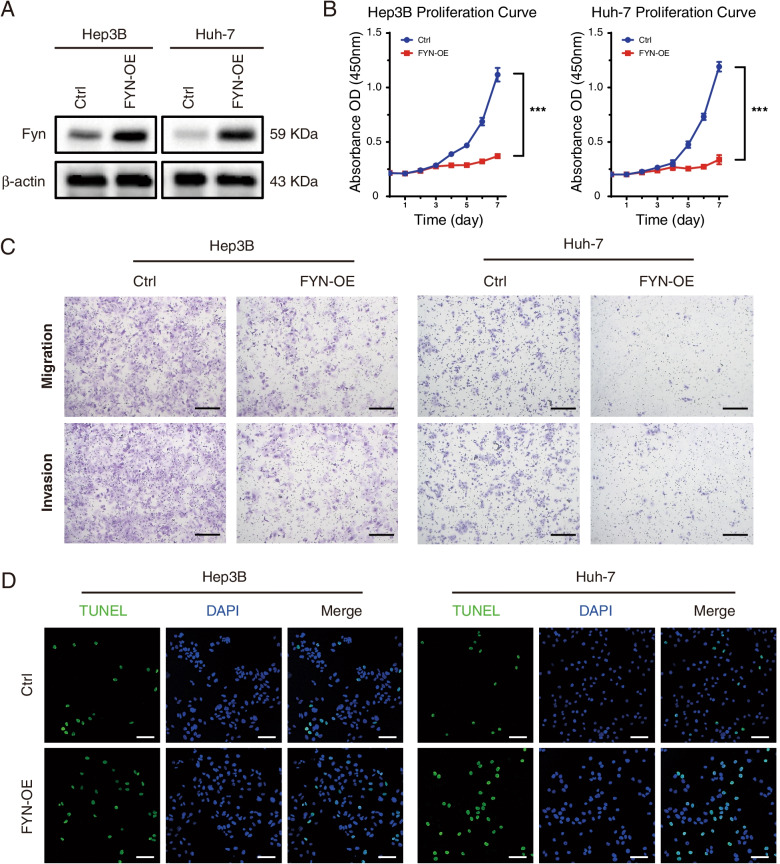
FYN-overexpression (OE) inhibited malignant behaviors and promoted the apoptosis in HCC cells. (A) FYN-OE lentivirus promoted the protein expression of FYN in Hep3B and Huh-7 cells, as shown western blot analysis. The blots cut prior to hybridization with antibody. (B) Effect of FYN-OE on the proliferation of Hep3B and Huh-7 cells determined by CCK-8 analysis. (C) Effect of FYN-OE on the migration and invasion of Hep3B and Huh-7 cells by determined by Transwell analysis. Scale bar: 200 μm. (D) Effect of FYN-OE on apoptosis of Hep3B and Huh-7 cells determined by TUNEL staining. Scale bar: 100 μm

It is generally believed that *FYN* is a proto-oncogene that plays a vital role in metabolism and physiological activities. However, its effect on HCC has not yet been clarified. To investigate whether *FYN* may act as a cancer suppressor gene, we then detected the migration and invasion abilities of FYN-overexpressing Hep3B and Huh-7 cells. As shown in Fig. 5 A-C and Supplementary Fig. 1 A-B, when compared with the control group, *FYN*-overexpression inhibited the proliferation and, also, migration, invasion abilities of Hep3B and Huh7 cells. Moreover, *FYN* overexpression could promote TUNEL-positive staining in HCC cells (Fig. 5D, Supplementary Fig. 1 C). Collectively, these results confirmed that the *FYN* might act as a tumor suppressor gene that participates the malignant behaviors of HCC cells.

### Overexpression of FYN suppressed the tumor growth in vivo

**Fig. 6:**
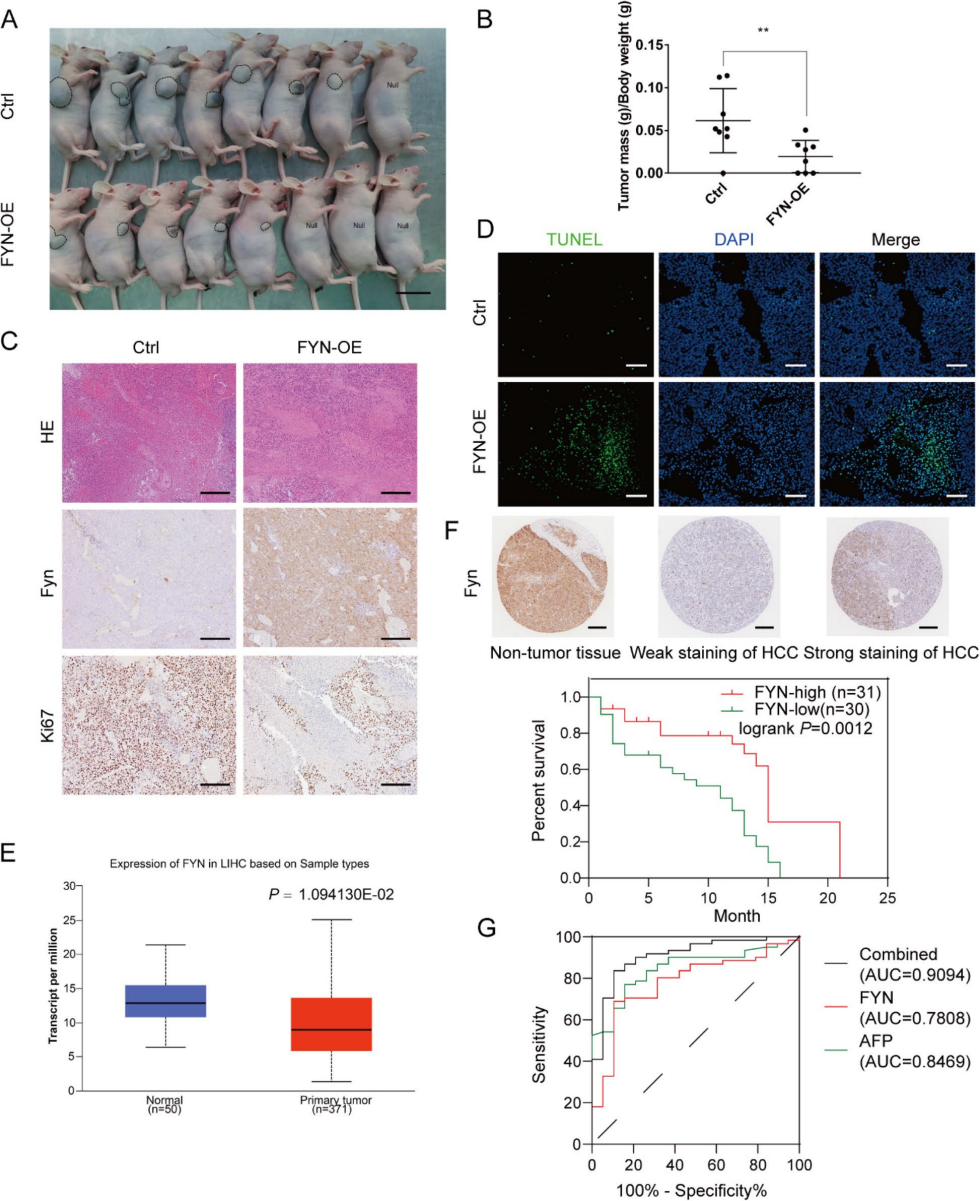
FYN-OE inhibited tumor growth in the HCC xenograft model. **A** Tumor size in the control (*n* = 8) and FYN-OE (*n* = 8) groups. Scale bar: 1 cm. **B** The ratio of the tumor mass to the bodyweight in the HCC xenograft model. **C** H&E and IHC staining of tumors in each group. Scale bar: 200 μm. **D** TUNEL staining of tumor in each group. Scale bar: 200 μm. **E** Expression of FYN in LIHC based on the sample types from the UALCAN database. **F** Tissue microarrays of Fyn IHC staining (upper panel). Survival curves of the relationship between FYN expression level and the patient's survival period. Red and green line represent FYN-high and low expression patient. **G** ROC curves of serum AFP, Fyn IHC score and combined condition

Next, we explored whether *FYN* exerted effects on the HCC xenograft model. As expected, the *FYN* overexpression group had significantly reduced the tumor volume and weight compared with the control group (Fig. 6 A-C).

Consistently, immunohistochemical staining and TUNEL assays assay of tumor tissues also confirmed that the *FYN* overexpression group exhibited a remarkable decrease of Ki67 positive cells along with an increasing number of apoptotic cells compared with the control group (Fig. 6 D, Supplementary Fig. 1 D-E).

From the UALCAN database, we found out that primary HCC tissues had a lower FYN expression than normal tissues (Fig. 6 E). Tissue microarrays containing HCC tissue samples (*n* = 61) and non-tumor tissue (*n* = 19) samples were examined for FYN expression by IHC. As shown in Fig. 6 F, Fyn high expression patients’ survival is significantly longer than Fyn low expression patients (logrank *P* = 0.0012). Furthermore, as shown in Fig. 6G, serum AFP AUC = 0.8469, while Fyn immunohistochemistry AUC = 0.7808, which is not better than serum AFP. When we unite FYN with AFP to identify the combined ROC curve, we found that combined curve AUC = 0.9094, better than single-end diagnosis AUC. But still need to expand the sample to make more rigorous verification.

## Discussion

Recent data showed that World Health Organization (WHO) announced that primary liver cancer caused 745,517 deaths worldwide, of which more than 50% were from China [1]. Due the over 120 million chronic hepatitis B virus (HBV) carriers at high risk of liver cancer in our country, achieving the early diagnosis, effective prevention and early, effective, and precise treatment of liver cancer is a long-term and arduous task [4, 15]. Better biomarkers for predictive and prognostic molecules for HCC are urgently required. The RNA-seq-expression and clinical information on HCC from the TCGA database were correlated by WGCNA, which allowed us to screen out genes closely related to the occurrence and development of cancer from the existing data.

In this study, through the identification of DEGs and WGCNA, we constructed a protein–protein network to determine the top 10 genes in the different modules by calculating the degree in Cytoscape, which identified three hub genes (*TGFBR3*, *COLEC10*, and *FYN*) that are intimately related to the survival outcome of HCC patients. Among these 3 genes, a lower expression level of *FYN* was significantly associated with both worse OS and DFS in the HCC patients. In contrast, CLOEC10 and TGFBR3 were only associated with worse OS. In addition, a previous study showed that TGFBR3 and COLEC10 were firmly related to the occurrence and development of HCC [16–18], but the biological functions of FYN, which is a member of the Src family of kinases (SFKs), have rarely been reported in HCC.

The SFKs include Src, Lyn, Fyn, Yes, Lck, Blk, and Hck, which play an essential role in regulating the growth and differentiation of eukaryotic cells [19, 20]. FYN is a 59 kDa protein whose carboxy terminus has extensive amino acid sequence homology with Src, but it is very different in a sequence of 81 amino acid residues at the amino terminus. The FYN protein is synthesized on the cytosolic polyribosome and is N-octadecylated, and then rapidly targets to the plasma membrane for palmitoylation. The corresponding sequences around Src Tyr416 and Tyr527 are conserved in FYN and, therefore, may be similarly regulated by phosphorylation. Double-acetylated FYN aggregates in caveolae-like membrane microdomains, which can interact with various other signal transduction molecules. FYN has various biological functions, including signal transduction through T cell receptors, regulation of brain function, and adhesion-mediated signal transduction. Changes in FYN levels in the corresponding target tissues may lead to better treatments for certain related diseases [20–23]. FYN is a proto-oncogene, but its role in tumors, especially hepatocellular carcinoma cells, has not been clarified, and its proposed roles are even contradictory.

Interestingly, when we overexpressed *FYN* in HCC cells, it seemed that *FYN* may acted as a tumor suppressor gene since it suppressed the migration and invasion ability and the proliferation of Hep3B and Huh-7 cells. Furthermore, *FYN* overexpression remarkably inhibited the colonization and growth of the tumors. Again, in the tissue microarrays of HCC patients, we found that the expression level of *FYN* in tumor tissues was lower than that in the adjacent tissues in most HCC patients, and the lower the expression level of *FYN* was, the worse was the prognosis of the patients. On the other hand, in the diagnosis of HCC, FYN is not superior to serum AFP. However, FYN and AFP were combined as HCC diagnostic criteria significantly better than AFP. However, because of the number of samples in this part, it may result in the resulting bias. In the future, the expansion of the sample quantity will further determine the FYN's diagnostic value of HCC, and whether it could be supplemental diagnostic criteria in clinical practice of HCC diagnosis.

## Conclusion

In conclusion, our study aimed to identify hub genes involved in HCC by WGCNA of data from the TCGA database and concluded that three functional genes (*TGFBR3*, *COLEC10*, and *FYN*) are highly differentially expressed in tumor and nontumor tissues. Finally, according to further study in vitro and in vivo studies, we preliminarily confirmed the anticancer effect of *FYN*, and it seems that *FYN* might be a promising biomarker use as a predictive and prognostic molecule for HCC. Moreover, the molecular mechanisms and the diagnostic value of *FYN* in for HCC patients should be further validated through a series of experiments.

## Supplementary Information

**Additional file 1: Supplementary Figure 1. **(A) (B) The quantitative analysis of Hep3B and Huh-7 cell migration and invasion in Ctrl and FYN-OE group. (C) The quantitative analysis of TUNEL staining of Hep3B and Huh-7 in Ctrl and FYN-OE group. (D) The quantitative analysis of Ki67 and FYN IHC staining of tumor section. (E) The quantitative analysis of TUNEL staining of tumor section. * *P* <0.05, ** *P* <0.01 and *** *P* <0.001 using two-tailed Student ’s t-tests. Experiments performed in triplicate, and data are presented as means ± SD 

## Data Availability

All gene expression profiles of LIHC are obtained from the TCGA database (portal.gdc.cancer.gov/projects/TCGA-LIHC) and GEO database (www.ncbi.nlm.nih.gov/geo/query/acc.cgi?acc=GSE25097). Other data used to support the findings of this study are within this article.

## Abbreviations

HCC: Hepatocellular Carcinoma
LIHC: Liver hepatocellular carcinoma
TCGA: The Cancer Genome Atlas
GEO: Gene Expression Omnibus
WGCNA: Weighted gene co-expression network analysis
PPI: Protein-protein interaction network
AFP: Alpha-fetoprotein
DEG: Differential Gene
CCK8: Cell Counting Kit 8
DMEM: Dulbecco's Modified Eagle's Medium
TUNEL: Terminal Dexynucleotidyl Transferase (Tdt)-Mediated dUTP Nick End Labeling
DAVID: The Database for Annotation, Visualization, and Integrated Discovery
KEGG: Kyoto Encyclopedia of Genes and Genomes
GO: Gene Ontology
OS: Overall Survival
DFS: Disease-free Survival
WHO: World Health Organization
HBV: Chronic Hepatitis B virus
