# Supplementary material for: A new finding in the key prognosis-related proto-oncogene FYN in hepatocellular carcinoma based on the WGCNA hub-gene screening trategy
Source: BMC Cancer. 2022 Apr 9;22:380. doi: 10.1186/s12885-022-09388-5 (PMC8994319; doi:10.1186/s12885-022-09388-5)
Supplement: Supplementary file 1 — Additional file 1: Supplementary Figure 1. (A) (B) The quantitative analysis of Hep3B and Huh-7 cell migration and invasion in Ctrl and FYN-OE group. (C) The quantitative analysis of TUNEL staining of Hep3B and Huh-7 in Ctrl and FYN-OE group. (D) The quantitative analysis of Ki67 and FYN IHC staining of tumor section. (E) The quantitative analysis of TUNEL staining of tumor section. * P <0.05, ** P <0.01 and *** P <0.001 using two-tailed Student ’s t-tests. Experiments performed in triplicate, and data are presented as means ± SD [file 12885_2022_9388_MOESM1_ESM.pdf]

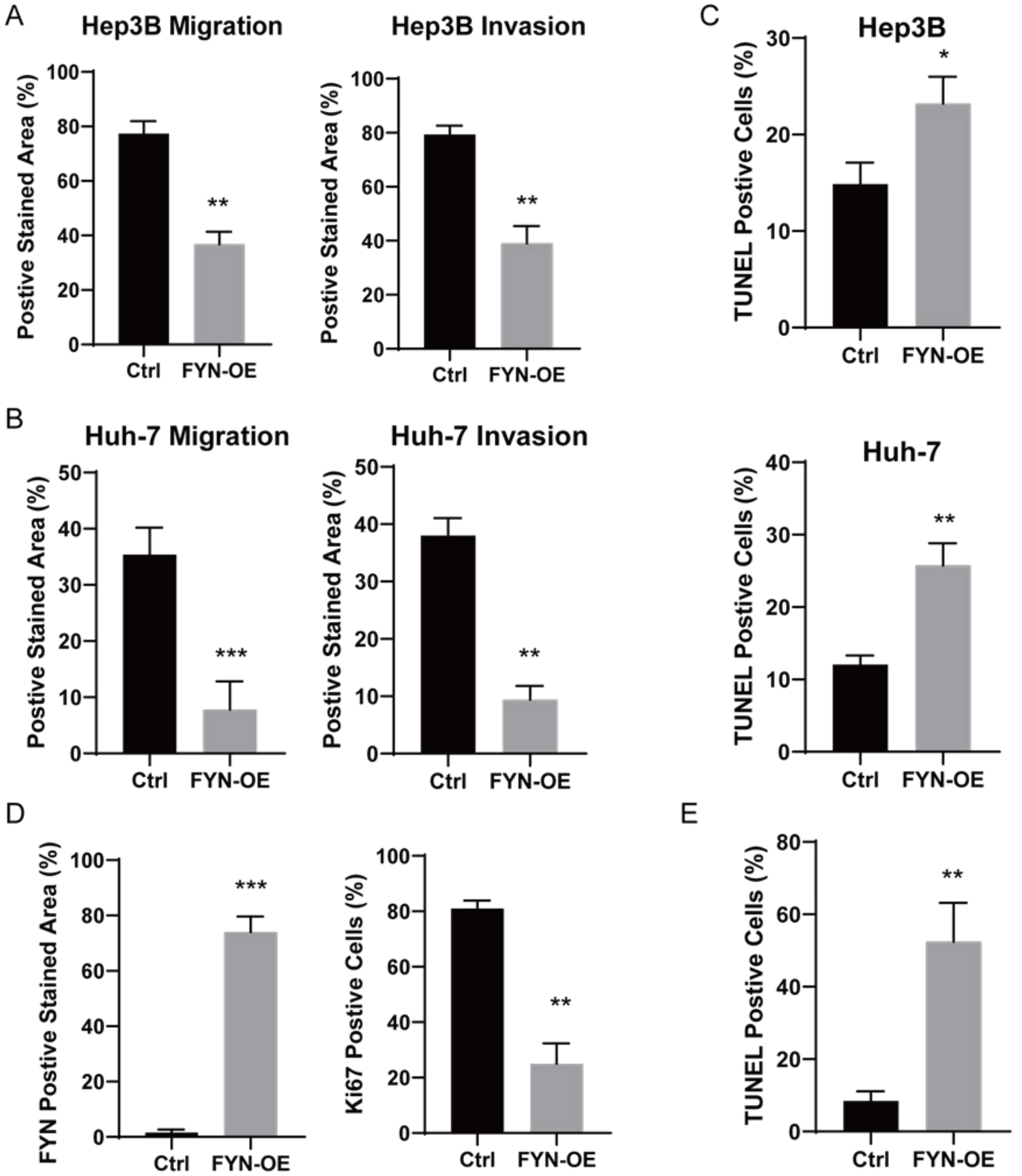

Supplementary Figure 1. (A) (B) The quantitative analysis of Hep3B and Huh-7 cell migration and invasion in Ctrl and FYN-OE group. (C) The quantitative analysis of TUNEL staining of Hep3B and Huh-7 in Ctrl and FYN-OE group. (D) The quantitative analysis of Ki67 and FYN IHC staining of tumor section. (E) The quantitative analysis of TUNEL staining of tumor section. * P <0.05, ** P <0.01 and *** P <0.001 using two-tailed Student 's t-tests. Experiments performed in triplicate, and data are presented as means ± SD.
